# Anti-HMG-CoA Reductase, Antioxidant, and Anti-Inflammatory Activities of* Amaranthus viridis* Leaf Extract as a Potential Treatment for Hypercholesterolemia

**DOI:** 10.1155/2016/8090841

**Published:** 2016-03-09

**Authors:** Shamala Salvamani, Baskaran Gunasekaran, Mohd Yunus Shukor, Noor Azmi Shaharuddin, Mohd Khalizan Sabullah, Siti Aqlima Ahmad

**Affiliations:** ^1^Department of Biochemistry, Faculty of Biotechnology and Biomolecular Sciences, Universiti Putra Malaysia (UPM), 43400 Serdang, Selangor, Malaysia; ^2^Faculty of Science and Natural Resources, Universiti Malaysia Sabah, Jalan UMS, 88400 Kota Kinabalu, Sabah, Malaysia

## Abstract

Inflammation and oxidative stress are believed to contribute to the pathology of several chronic diseases including hypercholesterolemia (elevated levels of cholesterol in blood) and atherosclerosis. HMG-CoA reductase inhibitors of plant origin are needed as synthetic drugs, such as statins, which are known to cause adverse effects on the liver and muscles.* Amaranthus viridis *(*A. viridis*) has been used from ancient times for its supposedly medically beneficial properties. In the current study, different parts of* A. viridis* (leaf, stem, and seed) were evaluated for potential anti-HMG-CoA reductase, antioxidant, and anti-inflammatory activities. The putative HMG-CoA reductase inhibitory activity of* A. viridis* extracts at different concentrations was determined spectrophotometrically by NADPH oxidation, using HMG-CoA as substrate.* A. viridis* leaf extract revealed the highest HMG-CoA reductase inhibitory effect at about 71%, with noncompetitive inhibition in Lineweaver-Burk plot analysis. The leaf extract showed good inhibition of hydroperoxides, 2,2-diphenyl-1-picrylhydrazyl (DPPH), nitric oxide (NO), and ferric ion radicals in various concentrations.* A. viridis* leaf extract was proven to be an effective inhibitor of hyaluronidase, lipoxygenase, and xanthine oxidase enzymes. The experimental data suggest that* A. viridis* leaf extract is a source of potent antioxidant and anti-inflammatory agent and may modulate cholesterol metabolism by inhibition of HMG-CoA reductase.

## 1. Introduction

Various forms of free radicals, such as alkoxy (RO) and nitric oxide (NO) radicals, are believed to contribute to the pathogenesis of chronic diseases. Hypercholesterolemia, which is characterized by the presence of high levels of cholesterol in the blood, is also believed to arise from oxidative stress. The enzyme, 3-hydroxy-3-methylglutaryl-coenzyme A (HMG-CoA) reductase, is a rate-limiting enzyme that catalyzes the conversion of HMG-CoA to mevalonate in the cholesterol biosynthesis pathway [1]. Statins competitively inhibit HMG-CoA reductase and are efficient in reducing the serum levels of low density lipoprotein (LDL) cholesterol. However, long-term consumption of statins causes serious side effects such as liver and muscles damage [2].

Edible medicinal plants with antioxidant and anti-inflammatory abilities can play a crucial role in the management of hypercholesterolemia [3]. Essentially, practicing healthy diets, including the consumption of desirable quantities of edible antioxidants, enables the body to neutralize radicals and offset damage associated with oxidative stress [4]. Flavonoids and phenolic acids are classes of polyphenolic substances with known antioxidant properties, including inhibition of oxidative enzymes, scavenging of free radicals, and induction of anti-inflammatory actions [5]. The advantages of using plants for medicinal purposes relate to their safety, availability, and economical benefits [6].

*Amaranthus viridis *L. (*A. viridis*), locally known as “bayam pasir,” belongs to the family of Amaranthaceae. It is a branched glabrous herb which is distributed in most of the tropical countries [7].* A. viridis* has been traditionally used to reduce labour pain and as an antipyretic in India and Nepal [8]. Other traditional usages are as analgesic, antiulcer, antirheumatic, antileprotic, and antiemetic agent [9]. It is also believed to treat eye diseases, psoriasis, eczema, asthma, and respiratory problems [10].

In the present study, the HMG-CoA reductase inhibitory activity of different parts of* A. viridis* (leaf, stem, and seed) was tested. Ferric thiocyanate (FTC), thiobarbituric acid (TBA), 2,2-diphenyl-1-picrylhydrazyl (DPPH) and nitric oxide (NO) scavenging activity, and ferric-reducing antioxidant power (FRAP) assays were used to measure the antioxidant activity while hyaluronidase, xanthine oxidase, and lipoxygenase inhibition assays were performed to determine the anti-inflammatory potential of* A. viridis* extract. The objectives of this study are to investigate anti-HMG-CoA reductase, antioxidant, and anti-inflammatory effects of* A. viridis*, focusing on the therapeutic potential relating to hypercholesterolemia.

## 2. Materials and Methods

### 2.1. Chemicals

All the chemicals and reagents used in this study were of analytical reagent grade and were purchased from Sigma-Aldrich and Merck.

### 2.2. Extraction of* A. viridis*

*A. viridis* was collected from various regions of Selangor, Malaysia. The plant was botanically identified and the plant voucher specimen was deposited in the Institute of Bioscience, Universiti Putra Malaysia. The plant was washed and separated into leaf, stem, and seed. The parts of* A. viridis* were air-dried and ground using a blender (Panasonic MX 8967) and subjected to methanol 50% (v/v) distillation for 48 h. After filtration, the extracts were isolated using a separatory funnel. The crude methanol extracts were then concentrated using a rotary evaporator (Heidolph) under reduced pressure at 40°C and freeze-dried at −40°C [11].

### 2.3. Enzyme Assay

HMG-CoA reductase inhibitory activities of* A. viridis* (leaf, stem, and seed) were determined based on spectrophotometric measurements. Each crude extract (50 *μ*g) was mixed with reaction mixture containing NADPH (400 *μ*M), HMG-CoA substrate (400 *μ*M), and potassium phosphate buffer (100 mM, pH 7.4) containing KCl (120 mM), EDTA (1 mM), and DTT (5 mM), followed by the addition of HMG-CoA reductase (2 *μ*L). The reactants were incubated at 37°C and absorbance was measured at 340 nm after 10 min. Simvastatin (Sigma, Missouri, US) was used as positive control and distilled water as negative control. The HMG-CoA reductase inhibition (%) was calculated using the following formula [12]:

(1) Inhibition  %=Δ  Absorbance  control−Δ  Absorbance  testΔ  Absorbance  control×100.

### 2.4. Kinetic Study

The inhibition mode of* A. viridis *leaf extract was determined using various concentrations of HMG-CoA reductase (0.3, 0.6, 0.9, and 1.2 mmol/L) and three different concentrations of* A. viridis* (0.05, 0.15, and 0.25 mg/mL). The assay was analyzed using Lineweaver-Burk plot analysis according to the Michaelis-Menten kinetics [12].

### 2.5. Total Phenolic Content (TPC)

The TPC of* A. viridis *extracts was determined according to Meda et al. [13] using Folin-Ciocalteu assay. Briefly,* A. viridis *extracts (100 mg) were dissolved in methanol (10 mL). The extracts (100 *μ*L), sodium carbonate (7.5% (w/v), 2 mL), and Folin-Ciocalteu reagent (tenfold dilution, 2.5 mL) were mixed, vortexed, and incubated at 40°C for 30 min. The absorbance was measured at 760 nm using a UV-visible spectrophotometer (Pharmaspec UV-1650 PC, Shimadzu, Japan). TPC of* A. viridis *extracts were expressed as mg gallic acid equivalents/g dry weight.

### 2.6. Total Flavonoid Content (TFC)

The TFC of* A. viridis *leaf extracts was determined according to Ismail et al. [14] using aluminium calorimetric method.* A. viridis *extracts (100 mg) were dissolved in methanol (10 mL). The extracts (100 *μ*L) were mixed with sodium nitrite (5% (w/v), 300 *μ*L) and incubated at room temperature for 5 min. Aluminium chloride (10% (w/v), 300 *μ*L) and sodium hydroxide (1 N, 2 mL) were then added followed by the addition of distilled water up to a total volume of 5 mL. The absorbance was measured using a UV-visible spectrophotometer (Pharmaspec UV-1650 PC, Shimadzu, Japan) at 510 nm. TFC of* A. viridis *extracts were expressed as mg rutin equivalents/g dry weight.

### 2.7. Antioxidant Activity of* A. viridis* Extracts

#### 2.7.1. Ferric Thiocyanate (FTC)

FTC assay was performed according to the method described by Ismail et al. [14].* A. viridis *extracts (4 mg) were dissolved in methanol (4 mL) and mixed with linoleic acid (2.5%, 4.1 mL), phosphate buffer (50 mM, pH 7, 8 mL), and distilled water (3.9 mL). The mixtures were kept in screw-cap vials which were placed in an oven maintained at 40°C in the dark. The mixtures (100 *μ*L) were added to ethanol (75%, 9.7 mL) and ammonium thiocyanate (30%, 100 *μ*L). Three minutes after the addition of ferrous chloride solution (2 × 10^2^ M in 3.5% hydrochloric acid, 100 *μ*L) to the reaction mixtures, the absorbance was measured at 500 nm using a UV-visible spectrophotometer (Pharmaspec UV-1650 PC, Shimadzu, Japan). The procedure was repeated (at 24 h interval) until the absorbance of the control sample reached its maximum value. Ascorbic acid and *α*-tocopherol were used as the standard antioxidants.

#### 2.7.2. Thiobarbituric Acid (TBA)

TBA assay on* A. viridis *extracts was carried out according to the method described by Hendra et al. [15]. The test was performed instantly after the control sample (from FTC test) reached the maximum absorbance value. Aqueous trichloroacetic acid (20%, 1 mL) and aqueous thiobarbituric acid (0.67%, 2 mL) were added to the sample solution (2 mL) acquired from the FTC test. The mixtures were incubated in a boiling water bath for 10 min. After cooling, the mixtures were spun at 3000 rpm for 20 min. Absorbance of the supernatants was read at 532 nm using a UV-visible spectrophotometer (Pharmaspec UV-1650 PC, Shimadzu, Japan). For both FTC and TBA assays, the antioxidant activity was expressed as % inhibition as in the following formula:

(2) $$\begin{array}{c} \%\text{ inhibition}=\frac{\left({Ab}_{c}-{Ab}_{s}\right)}{{Ab}_{c}}\times 100\%, \end{array}$$

where Ab_*c*_ is the absorbance of control and Ab_*s*_ is the absorbance of sample.

#### 2.7.3. 2,2-Diphenyl-1-picrylhydrazyl (DPPH) Radical Scavenging Activity

The free radical scavenging activity of* A. viridis *extracts was determined as described by Ismail et al. [14] using the DPPH assay.* A. viridis *extracts (100 *μ*L) at various concentrations (50, 100, 150, 200, 250, and 300 *μ*g/mL) were added to methanol (490 *μ*L) and DPPH methanolic solution (4 mg/100 mL, 390 *μ*L). The mixtures were then vortexed and allowed to stand at room temperature in the dark for 60 min. Absorbance of the mixtures was measured using a UV-visible spectrophotometer (Pharmaspec UV-1650 PC, Shimadzu, Japan) at 515 nm. Ascorbic acid and *α*-tocopherol were used as the standard antioxidants. Free radical scavenging activity of* A. viridis *extracts was expressed as % inhibition as in formula (2).

#### 2.7.4. Nitric Oxide (NO) Scavenging Activity

The NO scavenging activity of* A. viridis *extracts was determined according to the method of Tsai et al. [16].* A. viridis *extracts (60 *μ*L) at various concentrations (50, 100, 150, 200, 250, and 300 *μ*g/mL) were mixed with sodium nitroprusside (10 mM in phosphate buffered saline, 60 *μ*L) in a flat-bottomed microtiter plate and incubated in the light for 150 min at room temperature. An equal volume of the Griess reagent was added to the wells to measure the nitrite content. *A*_546_ was measured with a microtiter plate reader (Stat Fax 3200 Microplate Reader, Awareness Technology Inc., USA). Ascorbic acid and *α*-tocopherol were used as the standard antioxidants. The NO scavenging activity was expressed as % inhibition as in formula (2).

#### 2.7.5. Ferric-Reducing Antioxidant Power (FRAP) Assay

The ferric-reducing effect of* A. viridis *extracts was determined according to the method suggested by Yen and Chen [17].* A. viridis *extracts (1 mL) at various concentrations (50, 100, 150, 200, 250, and 300 *μ*g/mL) were mixed with potassium phosphate buffer (0.2 M, pH 6.6, 2.5 mL) and potassium ferricyanide (1 g/100 mL, 2.5 mL). The mixtures were then incubated at 50°C for 20 min. After incubation, trichloroacetic acid (10%) was added to stop the reaction. An equal volume of distilled water was added to the mixtures followed by the addition of ferum chlorate (0.1 g/100 mL, 500 *μ*L). The mixtures were then allowed to stand at room temperature for 30 min. *A*_700_ was determined with a UV-visible spectrophotometer (Pharmaspec UV-1650PC, Shimadzu, Japan). The procedures were performed in triplicate and repeated with the standard antioxidants (ascorbic acid and *α*-tocopherol). The antioxidant activity (%) of the samples in FRAP assay was expressed as reducing power (%) according to the following formula:

(3) $$\begin{array}{c} \text{Reducing power }\left(\;\%\;\right)=\frac{\left(Ab_{s}\times Ab_{c}\right)}{Ab_{s}}\times 100\%, \end{array}$$

where Ab_*c*_ is the absorbance of control and Ab_*s*_ is the absorbance of sample.

### 2.8. *In Vitro* Anti-Inflammatory Assay

#### 2.8.1. Hyaluronidase Inhibition Assay

The assay was carried out according to the method described by Yahaya and Don [18] with slight modifications. All the solutions were freshly prepared before the assay.* A. viridis *samples (5 mg) were dissolved in dimethylsulphoxide (250 *μ*L). The samples were prepared at various concentrations (100, 200, 300, 400, and 500 *μ*g/mL) by dissolving in sodium phosphate buffer (200 mM, pH 7). Hyaluronidase (4 U/mL, 100 *μ*L) was mixed with sample solution (25 *μ*L) incubated at 37°C for 10 min. The reaction was initiated with the addition of substrate, hyaluronic acid solution (0.03% in 300 mM sodium phosphate, pH 5.4, 100 *μ*L), and incubated at 37°C for 45 min. The undigested hyaluronic acid was then precipitated with acid albumin solution (bovine serum albumin (0.1%) in sodium acetate (24 mM), pH 3.8, 1 mL). After 10 min incubation at room temperature, *A*_600_ was measured using a spectrophotometer (XMA 1200V, Seoul, Korea). The absorbance measurement in the absence of enzyme was used as a control value. Quercetin was used as the positive control to verify the performance of the assay. The assay was performed in triplicate. The percentage of hyaluronidase inhibition was determined using the following formula:

(4) $$\begin{array}{c} \%\text{ Inhibition}=\frac{Ab_{s}}{Ab_{c}}\times 100\%, \end{array}$$

where Ab_*c*_ is the absorbance of control and Ab_*s*_ is the absorbance of sample.

#### 2.8.2. Lipoxygenase Inhibition Assay

Lipoxygenase inhibitory activity was performed according to the method suggested by Pin et al. [19] with slight modification.* A. viridis *samples (10 mg) were dissolved in dimethylsulphoxide (500 *μ*L). The samples were prepared at various concentrations (100, 200, 300, 400, and 500 *μ*g/mL) by dissolving in sodium phosphate buffer (100 mM, pH 8). Sodium phosphate buffer (100 mM, pH 8, 2.46 mL),* A. viridis *samples (10 *μ*L), and soybean lipoxygenase solution (167 U/mL, 20 *μ*L) were mixed and incubated at 25°C for 10 min. The reaction was initiated with the addition of substrate, sodium linoleic acid solution (10 *μ*L). The enzymatic conversion of sodium linoleic acid to (9Z,11E)-(13S)-13-hydroperoxyoctadeca-9,11-dienoate was measured by observing the change of absorbance at 234 nm for 6 min using a UV-vis spectrophotometer (Evolution 201, Madison, USA). A control was prepared by replacing samples (10 *μ*L) with mixture of sodium phosphate buffer (2.74 mL) and dimethylsulphoxide (25 *μ*L) into the quartz. Quercetin was used as the positive control to verify the performance of the assay. The assay was performed in triplicate. The percentage of lipoxygenase inhibition was determined according to formula (2).

#### 2.8.3. Xanthine Oxidase Inhibition Assay

Xanthine oxidase inhibitory activity was determined according to the method of Yahaya and Don [18], with slight modifications.* A. viridis *samples (10 mg) were dissolved in dimethylsulphoxide (500 *μ*L). The samples were prepared at various concentrations (100, 200, 300, 400, and 500 *μ*g/mL) by dissolving in potassium phosphate buffer (50 mM, pH 7.5). Potassium phosphate buffer (50 mM, pH 7.5, 2.38 mL),* A. viridis* samples (10 *μ*L), and xanthine oxidase solution (0.4 U/mL, 10 *μ*L) were mixed and incubated at 25°C for 10 min. The reaction was initiated by the addition of the substrate, xanthine solution (100 *μ*M, 100 *μ*L). The enzymatic conversion of xanthine to uric acid and hydrogen peroxides was measured at *A*_295_ using UV-vis spectrophotometer (Evolution 201, Madison, USA). A control was prepared by replacing samples (10 *μ*L) with a mixture of potassium phosphate buffer (2.39 mL) and dimethylsulphoxide (25 *μ*L) into the quartz. Quercetin was used as a positive control to validate the performance of the assay. The assay was performed in triplicate. The percentage of xanthine oxidase inhibition was determined according to formula (2).

### 2.9. IC_50_ Value Determination

The sample concentration providing 50% of inhibition (IC_50_) was determined through interpolation of linear regression analysis, with lower IC_50_ values suggesting higher antioxidant and anti-inflammatory activities, and vice versa.

### 2.10. Statistical Analysis

Data were expressed as mean ± SD from values determined in triplicate. One-way analysis of variance (ANOVA) and the accompanying Dunnett test (SPSS, version 19) were applied to compare values of* A. viridis *extracts with those of the positive controls, and IC_50_ was analyzed by using nonlinear regression.

## 3. Results

### 3.1. HMG-CoA Reductase Inhibitory Effect

**Figure 1:**
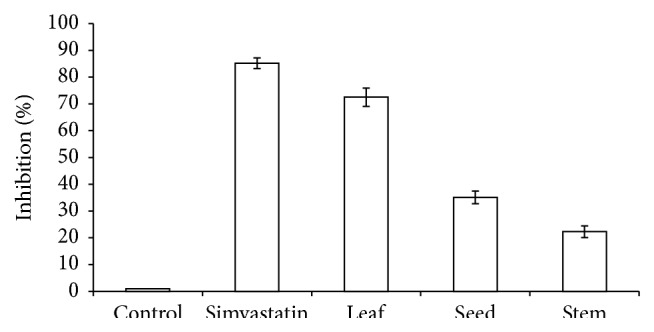
Inhibition of HMG-CoA reductase by* A. viridis* extracts. Distilled water was used as a negative control and simvastatin was used as a positive control. All data are presented as the mean ± SD of samples tested in triplicate.

**Figure 2:**
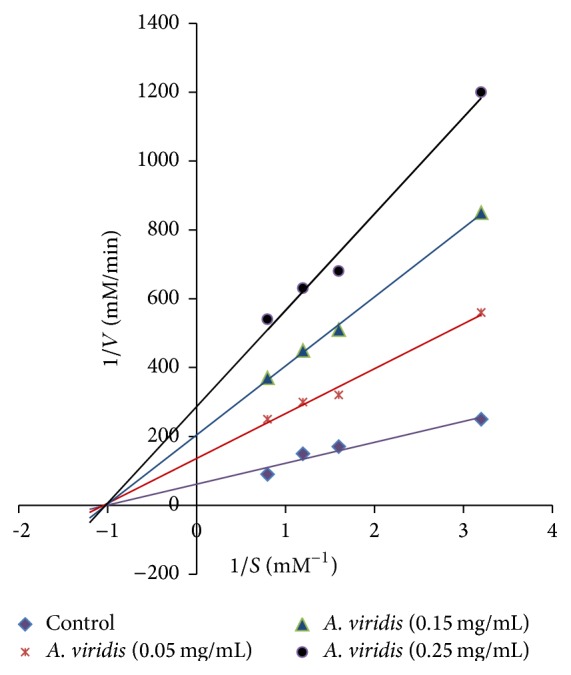
The Lineweaver-Burk plot analysis of HMG-CoA reductase in the presence of* A. viridis* leaf extract (0.05, 0.15, and 0.25 mg/mL) and HMG-CoA (0.3, 0.6, 0.9, and 1.2 mmol/L) at 340 nm.

*A. viridis *leaf, seed, and stem showed 72%, 35%, and 22% inhibitory effects, respectively, on HMG-CoA reductase activities (Figure 1). The leaf extract showed the highest inhibition and was further analyzed using Lineweaver-Burk plot analysis. Enzyme kinetics revealed noncompetitive inhibition on HMG-CoA reductase activity (Figure 2). Km value of HMG-CoA for HMG-CoA reductase was 1.0 mM. *V*_max_ value of the control was 0.0164. *V*_max_ values for* A. viridis *leaf extract at concentrations of 0.05, 0.15, and 0.25 mg/mL were 0.0074, 0.0049, and 0.0035, respectively.

### 3.2. Total Phenolic and Total Flavonoid Content

Total phenolic and total flavonoid content was determined using Folin-Ciocalteu and aluminium calorimetric assays, respectively.* A. viridis *leaf extract showed the highest phenolic and flavonoid content, which was approximately 85.83 mg GAE/g DW and 152.12 mg rutin equivalent/g DW, respectively. The results are summarized in Table 1.

### 3.3. Antioxidant Activities

**Figure 3:**
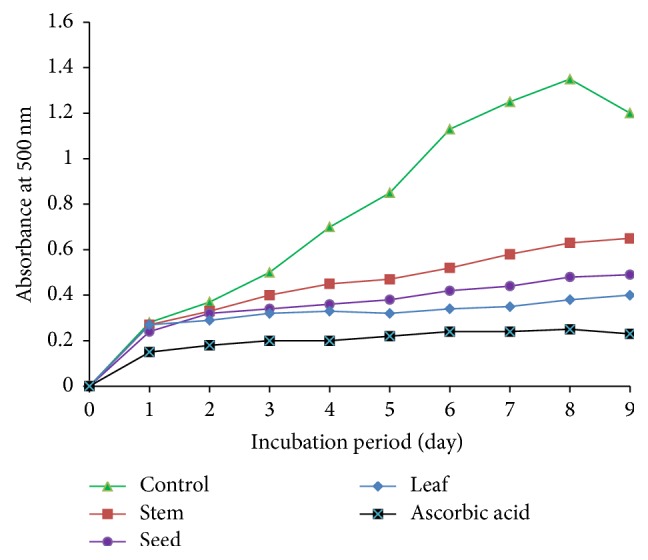
The inhibitory activity of* A. viridis *extracts on hydroperoxides in the ferric thiocyanate test.

**Figure 4:**
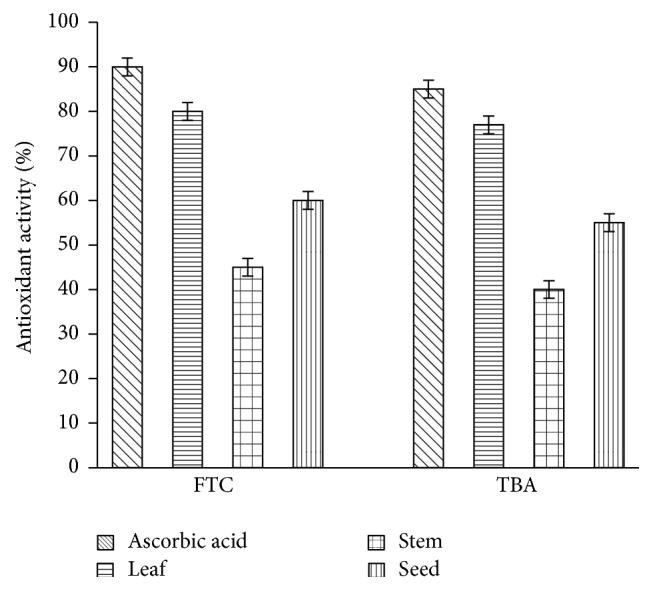
Total antioxidant activity assayed by ferric thiocyanate and thiobarbituric acid methods on day 9. The data represent the mean ± SD measurements of samples tested in triplicate.

The inhibitory activity of* A. viridis *extracts with respect to hydroperoxides was measured by the ferric thiocyanate assay (Figure 3). The leaf extract was found to have the best tendency to inhibit hydroperoxides compared to stem and seed. The results from FTC and TBA were almost similar in the sense that leaf extract was superior to stem and seed in terms of the potential to inhibit hydroperoxides (Figure 4).

**Figure 5:**
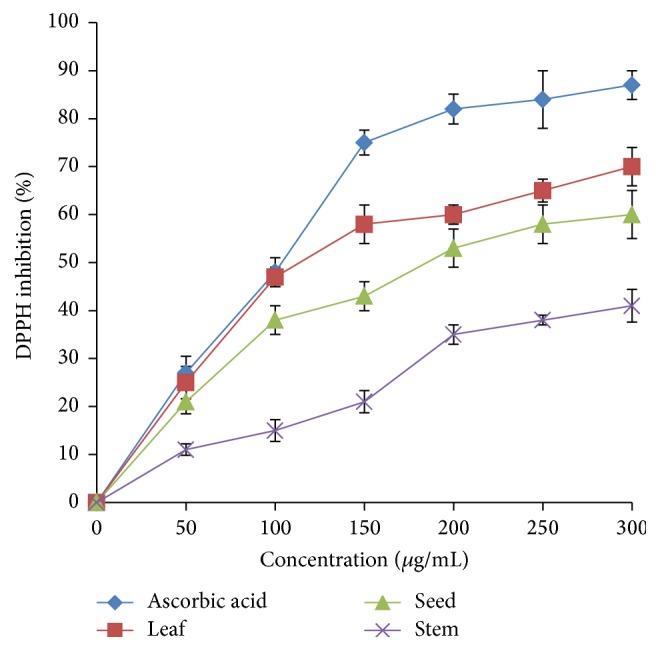
DPPH radical scavenging activity of* A. viridis *extracts at various concentrations. The data represent the mean ± SD measurements of sample tested in triplicate.

*A. viridis *extracts exhibited DPPH radical scavenging activity at various concentrations (Figure 5). All samples exhibited DPPH scavenging activity in a dose-dependent manner. Leaf extract achieved its highest inhibition at about 70%, followed by seed (58%) and stem (39%) at a concentration of 300 *μ*g/mL. IC_50_ values of leaf, seed, and stem were 115.74 *μ*g/mL, 189.21 *μ*g/mL, and >300 *μ*g/mL, respectively.

**Figure 6:**
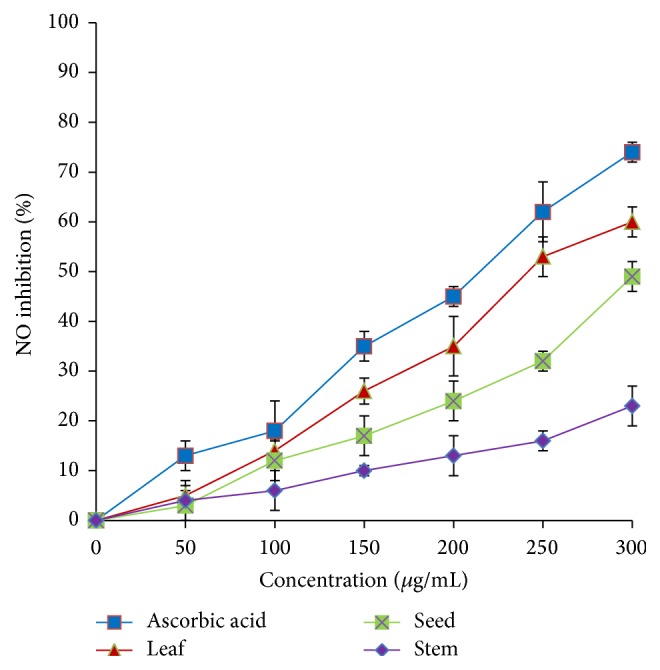
Nitric oxide scavenging activity of* A. viridis *extracts at various concentrations. The data represent the mean ± SD measurements of samples tested in triplicate.

All samples exhibited NO scavenging activity in a dose-dependent manner (Figure 6). The leaf extract exhibited the highest inhibition, about 60%, followed by seed (48%) and stem (22%) at a concentration of 300 *μ*g/mL. IC_50_ values of leaf, seed, and stem were 244.36 *μ*g/mL, 299.40 *μ*g/mL, and >300 *μ*g/mL, respectively.

**Figure 7:**
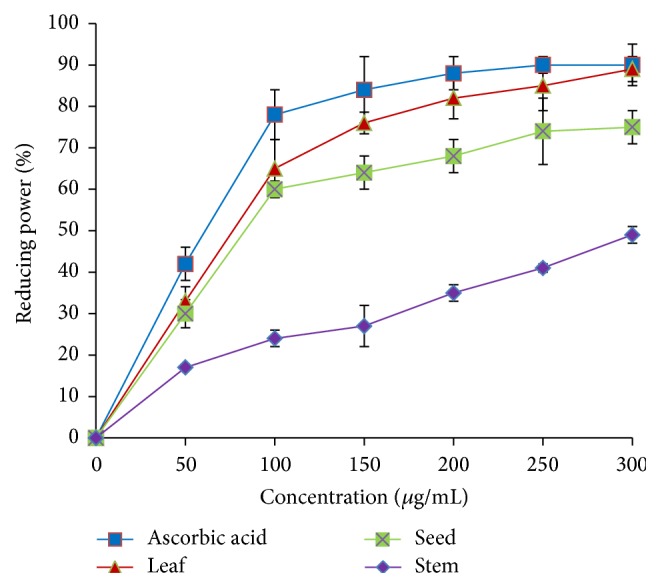
Ferric-reducing activity of* A. viridis *extracts at various concentrations. The data represent the mean ± SD measurements of samples tested in triplicate.

*A. viridis *extracts also exhibited satisfactory potential to reduce ferric ions at different concentrations (Figure 7). A drastic increase in the reducing power was observed at a concentration of 100 *μ*g/mL. The leaf extract exhibited the highest inhibition, about 85%, followed by seed (72%) and stem (48%) at a concentration of 300 *μ*g/mL. IC_50_ values of leaf, seed, and stem were 77.32 *μ*g/mL, 83.47 *μ*g/mL, and >300 *μ*g/mL, respectively.  Table 2 summarizes IC_50_ values of* A. viridis *extracts and standards for DPPH and NO radical scavenging and ferric-reducing activities.

### 3.4. Anti-Inflammatory Activities

**Figure 8:**
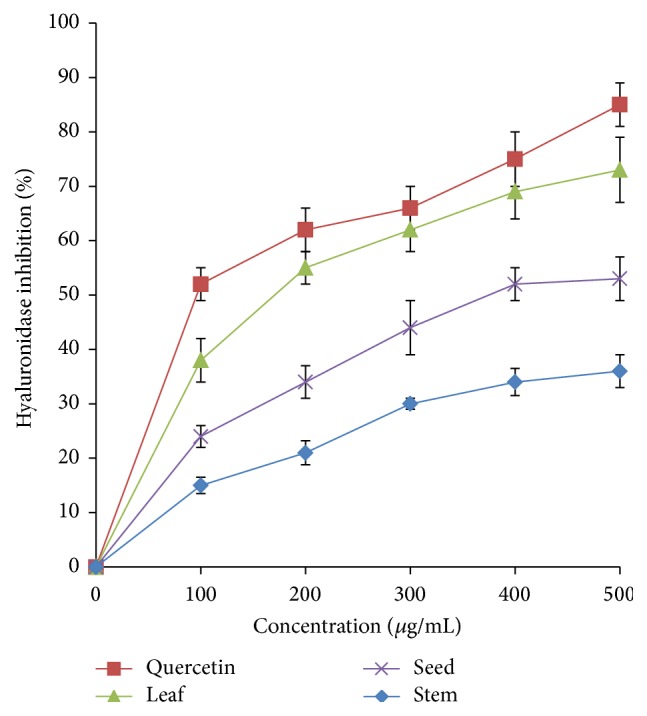
Hyaluronidase inhibitory activity of* A. viridis* extracts at various concentrations. The data represent the mean ± SD measurements of samples tested in triplicate.

The hyaluronidase inhibitory activity of the* A. viridis *extracts was investigated at different concentrations and was compared to quercetin (Figure 8). The inhibitory activity of the* A. viridis *extracts showed a gradual increase as the concentration increased. The leaf extract achieved about 70% inhibition at a concentration of 500 *μ*g/mL, followed by seed (50%) and stem (35%). IC_50_ values of leaf extract and quercetin were 186.23 *μ*g/mL and 101.64 *μ*g/mL, respectively.

**Figure 9:**
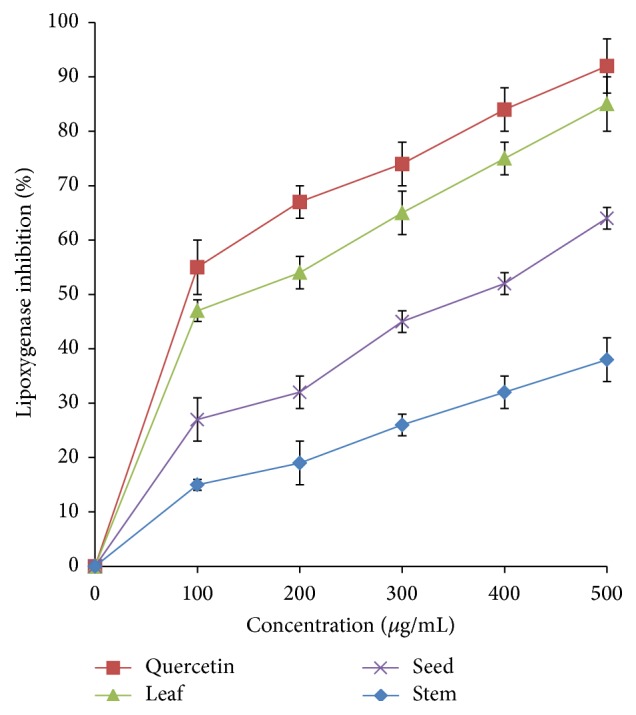
Lipoxygenase inhibitory activity of* A. viridis *extracts at various concentrations. The data represent the mean ± SD measurements of samples tested in triplicate.

Similarly, the investigation on lipoxygenase inhibitory activity revealed that* A. viridis *extracts inhibited the lipoxygenase enzyme in a dose-dependent manner. The highest inhibition, 82%, was exhibited by the leaf extract, followed by seed (60%) and stem (35%) at a concentration of 500 *μ*g/mL (Figure 9). IC_50_ values of leaf extract and quercetin were 151.59 *μ*g/mL and 98.36 *μ*g/mL, respectively.

**Figure 10:**
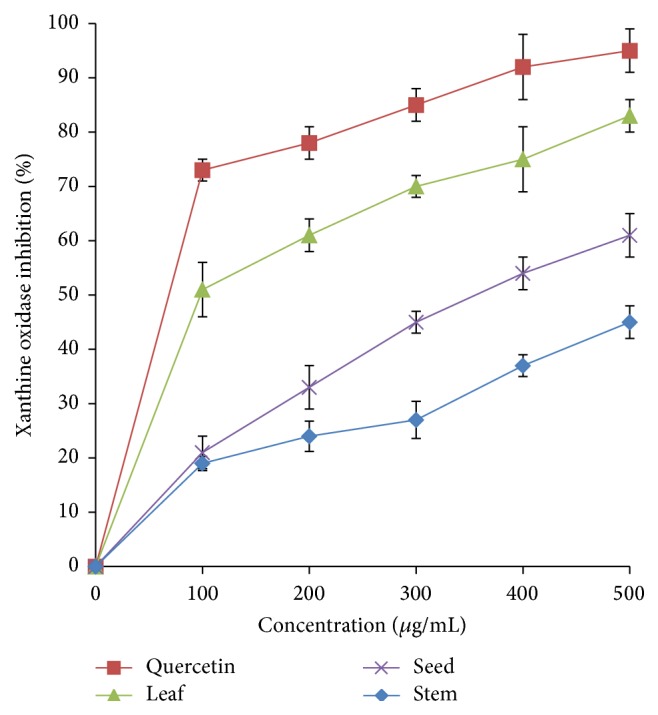
Xanthine oxidase inhibitory activity of* A. viridis *extracts at various concentrations. The data represent the mean ± SD measurements of samples tested in triplicate.

*A. viridis* extracts exhibited anti-xanthine oxidase activity in a concentration-dependent manner (Figure 10). The highest inhibition, approximately 80%, was achieved by the leaf extract at a concentration of 500 *μ*g/mL, followed by seed (58%) and stem (43%). IC_50_ values of leaf extract and quercetin were approximately 101.62 *μ*g/mL and 68.67 *μ*g/mL, respectively.  Table 3 summarizes IC_50_ values of* A. viridis *extracts and standards for hyaluronidase, lipoxygenase, and xanthine oxidase inhibition assays.

## 4. Discussion

Among the* A. viridis* extracts, the leaf extract showed the highest inhibition of 71% on HMG-CoA reductase activity. The inhibitory effect of* A. viridis* leaf extract is comparable to that of* Basella alba* leaf extract (74%) which was reported as a potent inhibitor of HMG-CoA reductase [11]. This observation is of great relevance since HMG-CoA reductase is the target enzyme for hypercholesterolemia. Plant extracts that suppress HMG-CoA reductase may possibly be used as hypocholesterolemic agent. Statins are synthetic drugs that competitively inhibit HMG-CoA reductase [20]. In contrast,* A. viridis* leaf extract was shown to inhibit HMG-CoA reductase in a noncompetitive manner. *V*_max_ value was found to decrease as the leaf concentration increased, suggesting that the substrate was unable to bind to HMG-CoA reductase. The inhibitor interacts with the enzyme or enzyme-substrate complex in which the binding changes the shape of the active site and prevents the formation of cholesterol. The anti-HMG-CoA reductase activity of the* A. viridis* leaf extract reflects its potential in cholesterol reduction.

The presence of significant phenolic and flavonoid contents suggests the potential use of the* A. viridis *leaf extract as an alternative medicine. Phenolic compounds describe a class of secondary metabolites with a wide range of pharmacological activities [21]. Indeed, various biological roles of phenolic acids have been reported. They play important roles in secretion of bile and reduction of lipids and cholesterol levels [22]. Phenolics and flavonoids exhibit a wide range of biological activities, including anti-inflammatory [23], antioxidant [24], antidepressant [25], and antiulcer [26] activities. The results of the current study on* A. viridis *leaf are comparable to those of a study by Hendra et al. [15], who found that the plant* Phaleria macrocarpa* possessed a high phenolic and flavonoid content.

Antioxidants are compounds that impede oxidation of substrates, and this is characterized by the potential to trap free radicals [27]. The main role that antioxidants play within the body systems rests on their ability to react with free radicals. Free radicals are compounds with unpaired electrons. The reaction eliminates the reactive properties of the radical [28]. With slight modification, the tests used in the current study are acknowledged to be reliable in investigating the antioxidant and anti-inflammatory potential of various substances [18, 29]. In many instances, the methods that have been utilized in determining the antioxidant activities are oriented towards the tendency of the tested compounds to scavenge free radicals or complex metal ions [30, 31]. We believe that the fact that these methods have been used in the current study highlights the reliability of the study.

The radical scavenging and reducing activities of* A. viridis *extracts appear to have been related mostly to the flavonoid content. The standard, ascorbic acid, gave higher inhibition rate compared to* A. viridis *extracts. This suggests that the rate of inhibition of* A. viridis *extracts may be improved by increasing the concentration in all cases. The measurement of inhibitory activities of* A. viridis *extracts with respect to hydroperoxides, using the FTC assay, suggests that* A. viridis* leaf extract effectively inhibits hydroperoxides. The* A. viridis *leaf and ascorbic acid standard showed low absorbance values compared to the negative control, suggesting high levels of antioxidant activity. The differences in the potential to inhibit hydroperoxides were further assayed by FTC and TBA methods on day nine when the negative control's absorbance decreases. The similarity in the results between FTC and TBA suggests the reliability of the results. In the context of toxicity, despite the fact that hydrogen peroxide is unreactive in the body systems, it may be toxic to cells, inhibiting cell functioning abilities by yielding hydroxyl radicals [32]. In this regard, elimination of hydrogen peroxide is considered a crucial antidetoxification process [33].

*A. viridis *leaf exhibited the DPPH radical scavenging ability at various concentrations, and the inhibition rate of* A. viridis *extract can be further improved by increasing the concentration. Similar to the potential in inhibiting hydroperoxides, leaf extract exhibited good inhibition of DPPH, considering that it compared favorably with ascorbic acid in terms of its ability to scavenge DPPH radical. The results revealed that* A. viridis* leaf has certain components possessing desirable hydrogen donation capacity that are well placed to scavenge the DPPH radicals which can be linked to the significant concentrations of flavonoid and phenolic acid, as well as the presence of hydroxyl groups [34]. Thaipong et al. [35] further explained that the antiradical activity of phenolic acids depends on the presence of phenolic hydroxyl hydrogen on the molecular structure, as well as on the tendency of phenoxyl radicals to stabilize resulting from the donation of hydrogen.

The data suggest that the leaf extract had good scavenging activity on the NO radical, in which the effectiveness increased in a dose-dependent manner. The leaf extract effectively inhibited ferric ions and was as effective as ascorbic acid in scavenging ferric ion radicals at a concentration of 300 *μ*g/mL. The performance of* A. viridis* leaf on NO and ferric ions inhibition has interestingly found concurring results; this finding is consistent with those reported earlier [21, 33]. Napoli and Lerman [36] have suggested that the inhibition of NO and ferric ion radicals is crucial in the management of hypercholesterolemia. The formation of these radicals can also result in pronounced vasomotor dysfunction which can further lead to atherosclerotic lesions [37, 38].

IC_50_ values (*μ*g/mL) of* A. viridis* extract revealed its good radical scavenging and reducing power activities. These results are comparable with those obtained using* Phaleria macrocarpa, *an antioxidant rich medicinal plant [15]. The* A. viridis* leaf extract exhibited a desirable ability to inhibit hydroperoxides, DPPH, NO, and ferric ions; this shows that it has a strong antioxidant capacity and could be helpful in treating the diseases caused by radicals, such as hypercholesterolemia and atherosclerosis.

The degradation of extracellular matrix plays an essential role in the development of various diseases possessing an inflammatory background [39]. Hyaluronidase is one of the most vital enzymes in this process and plays a major role in controlling the size and concentration of depolymerised hyaluronan chains, which are capable of altering the activities of many pathological processes. Hyaluronidase can cause increased endothelial permeability which can lead to atherogenesis [40]. The results from the current study suggested that the* A. viridis* leaf extract possessed high antihyaluronidase activity.

Lipoxygenases (LOXs) composed of nonheme iron-containing dioxygenases are important enzymes in leukotrienes biosynthesis, which have been believed to play a key role in the pathophysiology of inflammatory diseases [41]. Cipollone et al. [42] found that the LOXs expressions increased in the carotid atherosclerotic plaques of patients. It is suggested that LOXs are promising targets for therapeutic approaches focused on inflammation reduction in atherosclerotic plaques [43]. The* A. viridis* leaf extract was proven to be an effective inhibitor of lipoxygenase enzyme.

Xanthine oxidase, an oxygen free radical, has been commonly associated with being an important route in the oxidative injury to tissues, particularly ischemia-reperfusion [44]. Xanthine oxidase is involved in the metabolism of xanthine to uric acid. Uric acid plays a pathogenetic role in cardiovascular diseases; hyperuricemia is generally associated with deleterious effects on vascular function [45]. The inhibition of xanthine oxidase may exert therapeutic effects on impaired vascular function. The data from the present study suggest that the* A. viridis* leaf extract exhibited satisfactory potential as an inhibitor of the xanthine oxidase enzyme.

An examination of IC_50_ values (*μ*g/mL) of* A. viridis *extract on inhibition of the enzymes activities reveals its potential as an anti-inflammatory agent. Since leaf extract compares favorably with quercetin in inhibiting hyaluronidase, lipoxygenases, and xanthine oxidase, it may possess beneficial effects in protecting the vascular endothelial cells from oxidative stress, inflammation, and atherosclerosis.

## 5. Conclusion

*A. viridis* leaf extract appears to be a potent inhibitor of HMG-CoA reductase in a noncompetitive manner. The presence of phenolic and flavonoid content suggests that* A. viridis* can be used as an antioxidant in alternative medicine. Indeed,* A. viridis* leaf extract also exhibited good potential to reduce hydroperoxides, DPPH, NO, ferric ions, hyaluronidase, lipoxygenase, and xanthine oxidase at various concentrations. An examination of IC_50_ values (*μ*g/mL) of* A. viridis* leaf extract and standards on radical scavenging, reducing power, and enzymes inhibitory activities suggests its potential as an antioxidant and anti-inflammatory agent. Thus, in the management of hypercholesterolemia,* A. viridis* leaf extract appears to possess appreciable anti-HMG-CoA reductase activity and beneficial antioxidant and anti-inflammatory effects. Investigation in an* in vivo* model could further confirm the potential of* A. viridis* leaf extract in treating hypercholesterolemia.

## Figures and Tables

**Table 1:** Total phenolic and flavonoid content of different parts of* A. viridis*.

Sample	Total phenolic content^a^ (mg/g DW)	Total flavonoid content^b^ (mg/g DW)
Leaf	85.83 ± 1.56	152.12 ± 1.41
Stem	26.38 ± 0.34	50.97 ± 0.75
Seed	38.12 ± 0.89	81.36 ± 0.36

a: gallic acid equivalent; b: rutin equivalent. The analyses were performed in three replications.

**Table 2:** IC_50_ values (*μ*g/mL) of *A. viridis* extracts and standard on radical scavenging and reducing power activities.

Sample	DPPH	NO	FRAP
Ascorbic acid	105.03 ± 1.31	217.64 ± 0.79	63.49 ± 1.75
Leaf	115.74 ± 1.64	244.36 ± 2.15	77.32 ± 0.96
Stem	>300	>300	>300
Seed	189.21 ± 1.25	299.40 ± 1.26	83.47 ± 1.25

The analyses were performed in three replications.

**Table 3:** IC_50_ values (*μ*g/mL) of *A. viridis* extract and standard on inhibitory activities of inflammatory enzymes.

Sample	Hyaluronidase	Lipoxygenase	Xanthine oxidase
Quercetin	101.64 ± 0.89	98.36 ± 1.83	68.67 ± 0.65
Leaf	186.23 ± 1.31	151.59 ± 0.82	101.62 ± 1.29
Stem	>500	>500	>500
Seed	398.25 ± 2.33	394.52 ± 1.35	374.43 ± 2.15

The analyses were performed in three replications.
